# Cases of Ascites, Cured by Compression

**Published:** 1833-02

**Authors:** 

**Affiliations:** Surgeon to the King of Sardinia, and to the Celtic Hospital.


### ASCITES.

***Cases of Ascites, cured by Compression.*** **By Dr. FENOGLIO, Surgeon to the King of Sardinia, and to the Celtic Hospital.** *(Gaz. Med.)*

IT appears, from the testimony of MM. BRICHETEAU and HUSSON, that a radical cure may be effected, in certain cases of ascites, by means of compression, without danger and with considerable certainty. M. Bricheteau, it is true, found that, in some instances, the patient could not bear this mode of treatment, in consequence of the great difficulty of breathing to which it gave rise: in some cases, also, abdominal pains were caused, which led to a suspicion that peritoneal inflammation existed together with the dropsical effusion: but such cases were exceptions to the general result.

M. FENOGLIO compares ascites to hydrocele, and believes that, in most cases, in order to obtain a radical cure, a certain degree of inflammation of the serous membrane which is affected must be caused, for the purpose of leading to salutary adhesions, or that the artificial inflammation may effect a radical cure in some other manner, which is not yet understood. The two following cases are interesting in a double point of view, inasmuch as they confirm the efficacy of this simple, and too-little-employed, mode of treatment in a disease which frequently rebels against the best practice; and, on the other hand, they show the symptoms which may result from the employment of compression, and the means by which they may be relieved. M. Fenoglio agrees with those who have previously written on the subject, that compression should not be tried in patients who are either much debilitated, or who are labouring under some structural disease of the viscera, which acts as a permanent cause in the production of the dropsical effusion. In such instances, the effect of compression would be very doubtful. Compression succeeds in those cases of dropsy to which the term "essential" was formerly applied, and which are supposed to arise from defective absorption or increased exhalation.

Case I. *Ascites after premature Labour; Failure of internal Remedies. Compression; Peritonitis; Cure.*

A woman, of very nervous temperament, and of lively disposition, became pregnant, for the first time, at the age of twenty-two, and after great mental suffering, occasioned by discord in her family, she fell into labour at about the seventh month of utero-gestation. After her labour she became dropsical, and various remedies were tried in vain. When she applied to Dr. F., for the purpose of being tapped, she was very pale, but not much weakened or emaciated; she could also move about with facility, considering the enormous size of her belly; pulse natural; her respiration was only impeded at intervals, and she complained occasionally of a painful sensation about the diaphragm; the abdominal parietes were tense and thin. She had at this time been dropsical twelve months. Every care was taken to ascertain that there was no complication of any other disease, and that the patient was not again pregnant.

After the administration of two enemas, followed by a few days' rest, thirty-six pounds of a thick turbid fluid were drawn off by tapping. She willingly consented to any treatment that was likely to effect a radical cure, and, as her case appeared very favorable for a trial of compression, in consequence of its being one of simple dropsy, unattended by organic disease, it was tried in the following manner: The belly was surrounded by large compresses, which were kept on by a bandage applied so tight that the patient could not breathe with quite her usual freedom.

The second day from this "*tentative hasardeuse*," there was a more abundant flow of urine; but the peritoneum and viscera appeared to suffer from the compression. At the end of the third day, so much pain was produced, that a high fever followed, with shiverings, nausea, and vomiting. The desired inflammation was now clearly established; but, from an apprehension that this irritation, which had been designedly produced, might lead to disastrous consequences, if it were not confined within proper bounds, the compression was removed, emollient cataplasms were applied, and the patient was bled largely three times in twenty-four hours.

By this energetic treatment, the symptoms in a great measure subsided, and the patient slept for a short time. Nitre and digitalis were subsequently given, with the double intention of completely removing any disposition to inflammation that might still remain, and of causing a free flow of urine.

At the end of six days, there was no appearance of inflammation; the fever had subsided, and the patient was rapidly advancing to a state of convalescence. No relapse happened: the belly did not swell, and the urine was discharged in a natural quantity. The patient was afterwards troubled with severe palpitation of the heart, which yielded, after some time, to the daily use of valerian and assafœtida. In a month after the operation, she returned home perfectly cured. Four years have elapsed, and she remains in good health.

Case II. *Ascites without Organic Disease. Paracentesis performed five times; Compression; Symptoms of Irritation; Cure.*

A man cook of Count N., who had been much addicted to drinking and other kinds of debauchery, became dropsical at the age of thirty-seven. His habitual intemperance, no doubt, produced the disease. He had still a robust appearance. He had been tapped four times, and now applied to Dr. F. to have the operation again performed. At this time his countenance was pale; belly enormously distended; pulse regular, and natural in strength. He was first purged by enemas, and forty pounds of reddish-looking serum, without any disagreeable smell, were afterwards removed by tapping. The belly was carefully examined, but no visceral disease could be detected to contraindicate the employment of compression, the effect of which it was determined to try.

Compresses and bandages were applied, as in the previous case, and they were kept on until the appearance of inflammatory symptoms; but bleeding was not required in this case: it was merely necessary to remove the compression, and to apply emollient embrocations. A natural flow of urine was now established; the patient was speedily relieved of the dropsy; and three years have elapsed without any symptoms of relapse having occurred.

